# Exploring potential strategies to enhance memory and cognition in aging mice

**DOI:** 10.12688/f1000research.121922.3

**Published:** 2024-01-29

**Authors:** Shreevatsa Bhat M, Ramesh Babu M G, Anandh Dhanushkodi, Prof Kiranmai S Rai

**Affiliations:** 1 Division of Physiology,, DBMS, Manipal Academy of Higher Education, Manipal, Karnataka, 576104, India; 2 Department of Regenerative Medicine, Manipal Institute of Regenerative Medicine, Yelahanka, Bengaluru, Karnataka, 560065, India

**Keywords:** Aging, cognition, spatial memory, choline, and DHA, enriched environment, and HEK cell-conditioned media

## Abstract

**Background:**

Aging population is rapidly expanding worldwide, and age-related cognitive impairments prove detrimental for achieving a better productive and quality of life. Lack of effective therapies for age-related cognitive impairment focuses attention on developing preventive strategies, such as nutritional interventions, cell therapies and environmental manipulations. The objective of the present study was to explore the comparative benefits of potential memory-enhancing strategies like supplementation of choline (Ch) and docosahexaenoic acid (DHA) or administration of human embryonic kidney stem cell conditioned media (HEK-CM) or exposure to environmental enrichment (EE), that attenuates cognitive impairments in aging mice.

**Methods:**

Twelve-month-old CF1 male mice were subdivided [n=6/group] into normal aging control (NAC), saline vehicle control (SVC), Ch-DHA, EE, heat-inactivated HEK-CM (HIHEK-CM) and HEK-CM groups. Spatial working and reference memory were assessed using an eight-arm radial maze test and cognition using a novel object recognition test (NORT).

**Results:**

Spatial memory and cognition were decreased in normal aging mice. Aged mice exposed to dietary Ch-DHA or HEK-CM showed significant enhancement in spatial learning tasks, memory and cognition compared to the same in age-matched NAC mice. Ch-DHA and HEK-CM treated mice committed significantly lesser reference memory errors and attained a higher percentage of correct choices in spatial learning and memory tasks. Moreover, on testing for cognition in NORT, significantly higher number of visits to the novel object was observed in Ch-DHA supplemented and HEK-CM administered aging mice whereas HEK-CM and EE mice groups showed significantly greater number of visits to familiar object, when compared to same in age-matched NAC and HIHEK-CM groups, respectively.

**Conclusion:**

Supplementation of Ch-DHA and HEK-CM treatment strategies have a higher potential [~ 20—30%] for enhancing spatial learning, memory and cognition in normal aged mice, whereas exposure to EE seems to enhance only their short-term memory.

## Introduction

Normal aging is associated with a decline or functional deficits in multiple physiological domains, including cognitive functions. Cognitive abilities crucially determine successful/healthy aging and their impairment during aging specifically affects the quality of life and reduces productivity in elderly individuals. The growing burden of cognitive loss in rapidly increasing aging populations is shouldered not only by elderly patients and their family members but also by country/worldwide health care organizations. Although some mental functions such as general knowledge, numerical and verbal abilities are less affected during aging, other mental abilities like short-term memory, working memory, reasoning, processing speed, and executive functions weaken from middle age onwards or before. ^1^^,^^2^ Age-related learning and memory impairments are due to neuronal deficits that are associated with hippocampal damage. Age-related changes in cognitive ability are also attributed to altered hippocampal function. ^3^ All cognitive functions are not equally affected by aging, and it is also reported that functions like delayed recall of verbal information, ^4^ short-term recall, working memory ^5^ and spatial memory ^6^ decline with aging. Lack of effective disease-modifying therapies for age-related cognitive impairment seeks increasing attention on preventive strategies, such as nutritional interventions, cell therapies and environmental manipulations. Nutritional intervention has a major role in preventing/delaying deficits observed on neuronal function in aging such as cognitive ability and behavior. Important nutrients like choline (Ch) and docosahexaenoic acid (DHA) are required for normal brain function in humans and animals. Ch is derived from diet and by *de novo* synthesis in liver. ^7^ It is essential for membrane structural integrity, methyl group metabolism and neurotransmitter synthesis. ^8^ In rodents, maternal intake of Ch during gestation increases nerve cell proliferation and decreases apoptosis of fetal hippocampal neural progenitor cells, ^9^ enhances long-term potentiation in the hippocampus, and improves the auditory and visuospatial memory throughout their life. In adult rats, Ch supplementation improves cognitive abilities ^10^ and enhances the temporal memory. ^11^ DHA supplementation increases the level of synaptic proteins and membrane phospholipid in the hippocampal neurons ^12^ and enhances cognitive activities. ^13^ Several studies report that DHA deficiency may have an increased risk of developing cognitive disorders such as dementia and Alzheimer’s disease. ^14^^–^^16^ Cellular senescence is the restricted proliferation of cells, which contributes to aging of tissue and organism. ^17^ Further, currently, cell and cell-derived therapies are emerging as potential modes for treating various brain diseases/neurological disorders. Significant findings indicate that stem cell-based therapies potentiate functional recovery of neurological injury or neural disorders in animals. ^18^^–^^21^ Embryonic stem cells provide great potential for cell-based therapy in the field of regenerative medicine. ^22^^,^^23^ Alternatively, embryonic stem cells derived conditioned medium has been shown to be beneficial mainly through their chemical factors, for cell proliferation and tissue regeneration. ^24^^,^^25^ Moreover, studies also show that environmental enrichment (EE) impacts the brain positively by exposure to varying and stimulating physical and social surroundings. Increased rates of synaptic formation in the brain and improved activity have been observed when exposed to richer and stimulating surroundings. Studies in aged rats show that EE exposure results in permanent neuronal plasticity in the hippocampus and prevents age-associated impairments of spatial learning. ^26^ Daily exposure to EE in old rodents revealed its stimulating effect on neurogenesis possibly by increasing the survival rate of new neurons till their maturity. ^27^^,^^28^ Long-term exposure to EE in aged mice results in overall enhancement in cognitive ability. This proposes that long-term EE could provide cognitive stabilization. ^29^

Although these studies indicate the potential of each of the aforementioned therapies for improving cognitive functions and memory, no studies have explored comparing these beneficial strategies to identify the best in preventing normal age-associated decline in cognition and memory functions. Thus, the objective of the present study was to explore and identify the comparative potential memory-enhancing benefits of supplementation of Ch-DHA or administration of human embryonic kidney stem cell conditioned media (HEK-CM) or exposure to EE, in preventing normal age-associated decline in cognition and memory functions of normal aging CF1 mice. The benefits of using the CF1 mouse species as an animal model include its suitability for general multipurpose models, safety and efficacy testing, as well as these mice have been extensively employed in diverse neuroscience research studies worldwide. One of the key advantages lies in their genetic makeup, which bears a closer resemblance to human genes. ^30^

## Methods

### Animals

Approval for the study was obtained from Institutional Animal Care and Use Committee [No. IAEC/KMC/107/2014], MAHE, Manipal. All experiments were carried out in accordance with guidelines provided by the IAEC and CPCSEA, Government of India. A total of 66 middle-aged (12 to 15-months-old) CF1 male mice housed in Central animal house research facility, MAHE, Manipal, were used for the study. Experiment was conducted in 44 days including 30 days of intervention period and 14 days of behaviour analysis. During 44 days of experimental period [09/03/2019 to 22/04/2019], 4 to 6 mice were housed and bedded in standard polypropylene cages containing paddy husk as bedding material that was replaced every two days. Standard lab conditions, with temperatures ranging between 23±2°C, humidity (50±5%), and 12 hrs light-dark cycle were maintained for all the mice. Pellet feed with Ch content of 1 mg/kg, procured from VRK Nutritional Solutions [VRK’s “Scientist’s Choice” Laboratory Animal Diets, Pune] and water *ad libitum* were freely accessible to these mice. As expected, 10 to 15% of mortality was observed in the mice due to ageing.

### Experimental groups

Middle-aged male CF1 mice were randomly grouped [n=6/group] into normal aging control (NAC), saline vehicle control (SVC), Ch-DHA, HEK-CM, heat inactivated HEK-CM (HIHEK-CM) and EE groups. The NAC mice group remained undisturbed in the home cage throughout the 30 days interventional period of the experiment. The SVC group mice (included only for eight arm radial maze test) were fed equi-volume of saline for 30 days, Ch-DHA group mice were fed 45mg/kg body weight of Ch and 300mg/kg body weight of DHA for 30 days, HEK-CM group of mice were injected 100 μL of HEK-CM and HIHEK-CM group mice were injected 100 μL of heat inactivated HEK-CM through intravenous (tail vein) injections for 5 days, specifically on the 1 ^st^, 6 ^th^, 11 ^th^, 16 ^th^ and 21 ^st^ day of the experimental period and EE group of mice were exposed to enriched environment for 30 days.

### HEK-CM derivation

Cryopreserved 293T cells, which are derived from human embryonic kidney (HEK), were rapidly thawed at 37°C. HEK cells from a cryovial which contained dimethyl sulfoxide were transferred to a centrifuge tube (15 mL) and 5 mL of HEK media (Dulbecco’s modified Eagle’s medium-high glucose) was added along with 10% fetal bovine serum, 1% penicillin-streptomycin, 1% nonessential amino acids and 1% of 200 mmol/L L-glutamate and centrifuged at 1800 rpm for 5 min. After discarding the supernatant, the pellets were re-suspended in 3 mL of HEK media and the cell suspension was then transferred into 25-cm ^2^ culture flasks to incubate with 5% CO _2_ at 37°C for 24 hrs. The HEK cells were grown until they attained a confluency of 70 to 80%. HEK-CM, the conditioned media in which the HEK cells were grown was stored at -80°C for further use. HIHEK-CM was used as the vehicle control for HEK-CM and was prepared by subjecting HEK-CM to heat inactivation at 60°C in a water bath for 30 minutes, followed by cooling to room temperature. This process was employed to ensure that HIHEK-CM has no cognitive effects, as the heat treatment inactivates all the protein constituents of HEK-CM.

### Environmental enrichment

For the enriched condition large wire-mesh cages (70 × 70 × 45 cm) were provided with various objects (toys), which were changed daily. These consisted of small mazes, ladders, running wheels, swings, plastic and metal cubes, spheres, cylinders, and trays of small objects, that could be carried about by the mice.

### Eight-arm radial maze test

The eight-arm radial maze is an elevated plexiform maze placed 80 cm above the floor. It consists of an octagonal central platform from which equally spaced eight arms (each arm is 42 cm long, 11.4 cm high, and 11.4 cm wide) radiate and has a video monitor attached to a computer. After 30 days of treatment, mice were semi-starved for 2 days to reduce their body weight up to 85% and then subjected to eight arm radial maze test for 10 days, which consisted of 2 days of habituation phase followed by 4 days each of acquisition and retention phases.

During the habituation/orientation phase, all the eight arms were baited with food pellets and mice were allowed to orient and get habituated to the maze during two trials per day carried out for 2 days.

Acquisition phase of a spatial task is conducted following habituation in the radial maze. During this phase, bait of food pellets was placed only in four arms. Prior to each trial and each session, the maze was wiped with 50% ethanol to avoid any olfactory cues. The mice were placed in the centre of the maze and allowed to explore the maze freely. The mice were trained to take the food from baited arm without making a re-entry into the already visited arm. The trial was ended when the mice have taken the food from all four baited arms or after 5 min if mice did not visit the baited arms. During the trial, the animal’s performance was monitored and the number of entries into the arms were noted. Each mouse was given two trials per day for 4 days. The performance of the animal was scored by calculating the percentage of correct responses (a correct entry is the animal’s number of first visits to the baited arms) divided by the total number of entries made by the animal. Re-entries into previously visited baited arms were counted as working memory errors and entries into the unbaited arms were taken as reference memory errors.

Retention phase of spatial task in the radial maze: Subsequent to learning/acquisition phase, mice were retained in their individual cages for 4 days without any training. In order to assess the retention of the learned/acquired task, the performance of mice in the radial maze was again assessed for a single trial on the 4 ^th^ day. The experimental protocol remained same as that for the acquisition test.

### Novel object recognition test (NORT)

The cognitive functions of mice were assessed by using NORT. Rodents have an innate tendency to visit the novel objects repeatedly than to the familiar objects. Two round plastic container boxes filled with sand were used as familiar objects, and a wooden cube box with different shape and color was used as novel object. The objects were positioned in the center of the open field, with equal distance separating them from one another and from the sides of the field. Number of visits to a novel object gives a behavioral measure of retention of memory and discriminating ability between familiar and novel objects, thus revealing their cognitive and hippocampal function. The test was done as a new one-trial NORT method. In the habituation phase, animals were allowed to orient and habituate in an open field for 30 min. Mice were retained in their home-cage for 5 min after the 30 min habituation phase. Subsequently, during the acquisition phase, mice were exposed to two identical objects and allowed to explore and get familiarized with the objects for 5 min. Subsequently, mice were placed back in their respective cages. During the test phase [one day after the acquisition phase], mice were again exposed to the open-field arena with one familiar object and a novel object, for 5 min. The test phase was video monitored, and the number of visits by each animal to the familiar and novel objects were marked manually from the monitor and then counted. Only the active contact of animal with its nose, mouth or paws to the objects was considered as the number of visits. The accidental touch, such as if animal was backing into the object or bumping the object accidentally as it passed was not included for scoring. To remove any olfactory clues, the test arena and the objects were cleaned with 70% alcohol before placing a successive mouse for the test.

### Statistics

Data were presented as mean ±SEM and one-way analysis of variance [ANOVA] with Bonferroni's post-hoc test were used to compare the treatment effects between the groups, and a value of *p*< 0.05 was considered as statistically significant. SPSS (RRID: SCR_002865) was used for statistical analysis.

## Results

**Figure 1.**
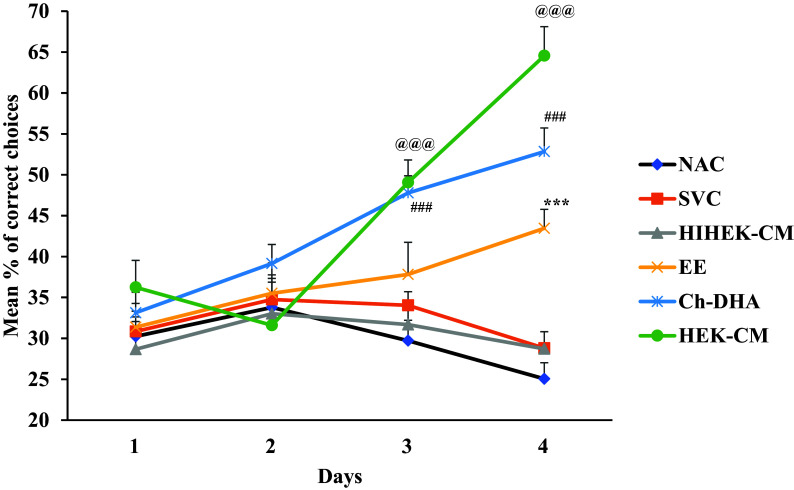
Graphical representation of performance of mice groups as a function of trial days during learning phase in the eight-arm radial maze task. Values represent mean ±SEM percentage of correct choices along with Bonferroni post-hoc test *p*-values. Human embryonic kidney stem cell conditioned media (HEK-CM) and choline-docosahexaenoic acid (Ch-DHA) mice vs heat inactivated HEK-CM (HIHEK-CM) or saline vehicle control (SVC) or normal ageing control (NAC) mice made significantly more correct choices on 3 ^rd^ and 4 ^th^ day of trials ^@@@^ and ^###^ *p*<0.001 respectively, whereas environmental enrichment (EE) exposed mice vs NAC mice made significantly more correct choices on 4 ^th^ day of trial. *** *p*<0.001.

**Figure 2.**
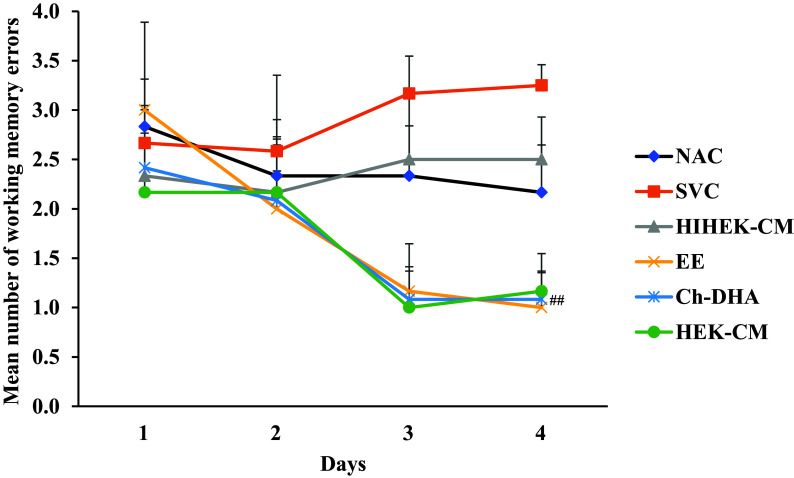
Graphical representation of performance of mice groups as a function of trial days during learning phase in the eight-arm radial maze task. Values represent mean ±SEM numbers of working memory errors, along with Bonferroni post-hoc test *p*-values. Choline-docosahexaenoic acid (Ch-DHA) mice vs saline vehicle control (SVC) mice made significantly less numbers of working memory errors on 4 ^th^ day of trial. ^##^ *p*<0.01 **.**

**Figure 3.**
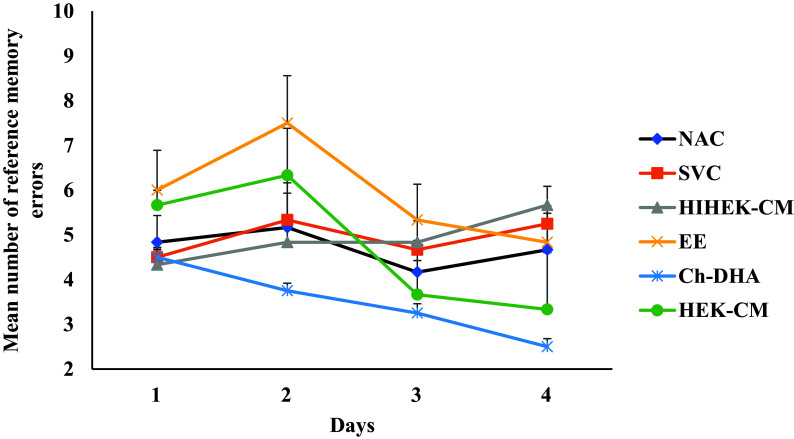
Graphical representation of performance of mice groups as a function of trial days during learning phase in the eight-arm radial maze task: Values represent mean ±SEM numbers of reference memory errors along with Bonferroni post-hoc test *p*-values. Human embryonic kidney stem cell conditioned media (HEK-CM) and Choline-docosahexaenoic acid (Ch-DHA) mice made slightly fewer mean numbers of reference memory errors on 3 ^rd^ and 4 ^th^ day of trials when compared with all other groups.

**Figure 4.**
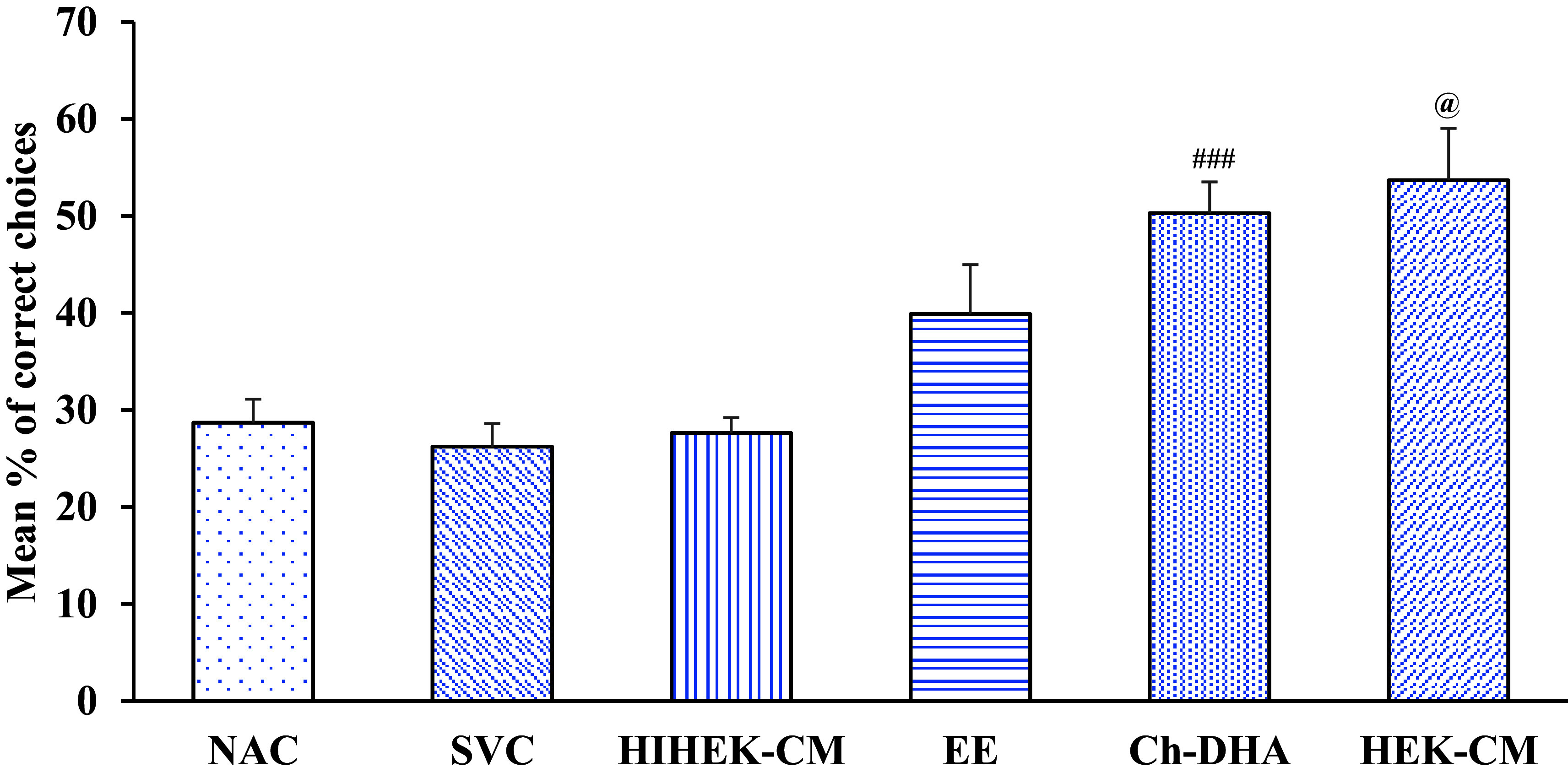
Graphical representation of performance of mice groups during retention phase in the eight-arm radial maze task: Values represent mean ±SEM percentage of correct choices along with Bonferroni post-hoc test *p*-values. Choline-docosahexaenoic acid (Ch-DHA) and human embryonic kidney stem cell conditioned media (HEK-CM) mice vs saline vehicle control (SVC) or normal ageing control (NAC) and heat inactivated HEK-CM (HIHEK-CM) mice respectively made significantly more percentage of correct choices during retention phase. ^###^ *p*< 0.001 and ^@^ *p*< 0.05.

Aging mice supplemented with Ch-DHA, or administered with HEK-CM showed significantly increased mean % of correct choices ( *p*< 0.001) indicating enhanced spatial learning during both the 3 ^rd^ and 4 ^th^ day of training whereas those mice exposed to EE showed significantly enhanced spatial learning ( *p*<0.001) only on fourth day of training relative to same in age-matched SVC, HIHEK-CM, and NAC mice ( Figure 1, Table 1). During the initial days of training, mice from all groups made random re-entries into arms already visited. As the training continued, although aging mice exposed to Ch-DHA or HEK-CM or EE, showed progressive decrease in working memory errors, they were significantly lower ( *p*<0.01) only in Ch-DHA supplemented mice compared to the same in age-matched SVC ( Figure 2, Table 2). However, when compared with aging mice exposed to Ch-DHA, HEK-CM and EE group of mice, NAC mice and HIHEK-CM mice groups committed more working memory errors throughout training, indicating poor learning. Moreover, although the mean number of reference memory errors committed by Ch-DHA and HEK-CM mice were lesser during the 3 ^rd^ and 4 ^th^ day of training, no significant difference in reference memory errors was observed when compared to the same in age-matched NAC mice groups ( Figure 3, Table 3). However, Ch-DHA and HEK-CM mice attained a significantly higher percentage ( *p*<0.001 and *p*<0.05, respectively) of correct choices during the retention test. In contrast, NAC, HIHEK-CM mice showed significant impairment in the retention of spatial tasks ( Figure 4, Table 4).

**Table 1.** Spatial memory performance of mice groups during learning phase in the eight-arm radial maze task.

Groups (n=6 / group)	Percentage of correct choices during learning phase Mean ± SEM			
	Day 1	Day 2	Day 3	Day 4
**NAC**	30.23 ±3.08	33.78±3.98	29.71±2.48	25.05±1.97
**SVC**	30.83±1.78	34.75±2.58	34.03±1.67	28.80±2.01
**HIHEK-CM**	28.66±3.39	33.01±3.85	31.68±2.17	28.71±0.62
**EE**	31.36±4.26	35.50±3.72	37.81±3.92	43.45±2.32 ^***^
**Ch-DHA**	33.13±1.14	39.15±2.33	47.78±2.09 ^###^	52.83±2.89 ^###^
**HEK-CM**	36.23±3.29	31.60±2.78	49.06±2.74 ^@@@^	64.55±3.56 ^@@@^

**Table 2.** Spatial working memory errors by mice groups during learning phase in the eight-arm radial maze task.

Groups (n=6 / group)	Number of working memory errors Mean ± SEM			
	Day 1	Day 2	Day 3	Day 4
**NAC**	2.83±0.47	2.33±1.02	2.33±0.84	2.16±0.47
**SVC**	2.66±0.38	2.58±0.32	3.16±0.38	3.25±0.21
**HIHEK-CM**	2.33±0.66	2.16±0.47	2.50±0.34	2.50±0.42
**EE**	3.00±0.89	2.00±0.73	1.16±0.47	1.00±0.36
**Ch-DHA**	2.41±0.41	2.08±0.30	1.08±0.32	1.08±0.27 ^##^
**HEK-CM**	2.16±0.60	2.16±0.54	1.00±0.36	1.16±0.30

**Table 3.** Spatial reference memory errors by mice groups during learning phase in the eight-arm radial maze task.

Groups (n=6 / group)	Number of reference memory errors Mean ± SEM			
	Day 1	Day 2	Day 3	Day 4
**NAC**	4.83±0.60	5.16±1.13	4.16±0.47	4.66±0.91
**SVC**	4.50±0.22	5.33±0.60	4.66±0.21	5.25±0.40
**HIHEK-CM**	4.33±0.33	4.83±1.32	4.83±0.47	5.66±0.42
**EE**	6.00±0.89	7.50±1.05	5.33±0.80	4.83±0.65
**Ch-DHA**	4.50±0.18	3.75±0.17	3.25±0.21	2.50±0.18
**HEK-CM**	5.66±0.33	6.33±1.05	3.66±0.76	3.33±0.80

**Table 4.** Correct choices made by mice groups during spatial memory retention phase in the eight-arm radial maze task.

Groups (n=6 / group)	Percentage of correct choices during retention phase Mean ± SEM
**NAC**	28.68±2.42
**SVC**	26.21±2.37
**HIHEK-CM**	27.61±1.59
**EE**	39.88±5.10
**Ch-DHA**	50.28±3.22 ^###^
**HEK-CM**	53.68±5.34 ^@^

**Figure 5.**
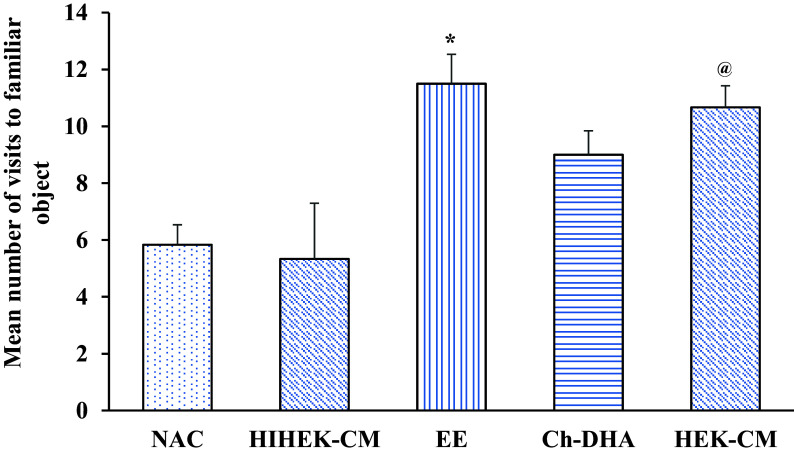
Graphical representation of tendency of mice groups to visit familiar object in NORT: Values represent mean ±SEM number of visits to the familiar object along with Bonferroni post-hoc test *p*-values. Environmental enrichment (EE) and human embryonic kidney stem cell conditioned media (HEK-CM) mice vs normal ageing control (NAC) and heat inactivated HEK-CM (HIHEK-CM) mice respectively made significantly greater number of visits to familiar object * and ^@^ *p*<0.05.

**Figure 6.**
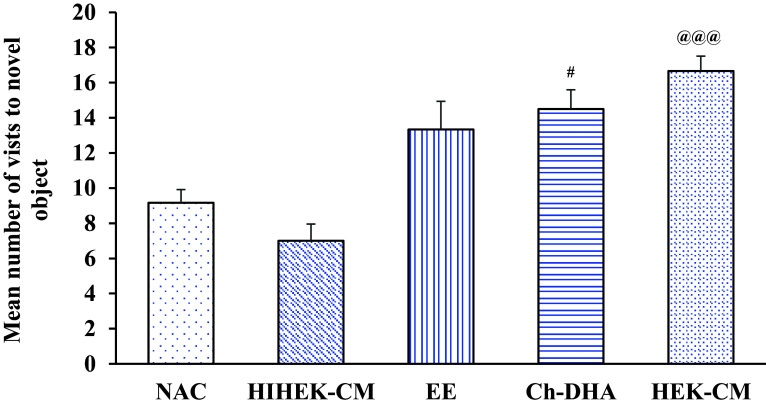
Graphical representation of tendency of mice groups to visit novel object in NORT: Values represent mean ±SEM number of visits to the novel object along with Bonferroni post-hoc test *p*-values. Human embryonic kidney stem cell conditioned media (HEK-CM) and choline-docosahexaenoic acid (Ch-DHA) mice vs heat inactivated HEK-CM (HIHEK-CM) or normal ageing control (NAC) mice respectively made significantly greater number of visits to novel object. ^@@@^ *p*<0.001 and ^#^ *p*<0.05

When aging mice supplemented with Ch-DHA, or exposed to HEK-CM or EE were assessed for cognition in NORT, significant differences in visits to the familiar object, were observed in HEK-CM and EE groups when compared to NAC and HIHEK-CM groups ( *p*<0.05). Moreover, aging mice supplemented with Ch-DHA also showed slightly higher number of visits to familiar object compared to NAC mice but it was not significant ( Figure 5, Table 5). When the number of visits to novel objects was compared between all groups, HEKCM aging mice showed significantly higher preference ( *p*<0.001) with higher number of visits to the novel object as compared to the same in age-matched NAC and HI-HEKCM mice groups. Whereas, Ch-DHA supplemented aging mice showed higher preference at lower significant levels ( *p*< 0.05) to the novel object as compared to the same in age-matched NAC mice group ( Figure 6, Table 6).

**Table 5.** Cognitive performance of mice groups showing visits to familiar object in the NORT.

Groups (n=6 /group)	Number of visits to familiar object Mean ± SEM
**NAC**	5.83±0.70
**HIHEK-CM**	5.33±0.84
**EE**	11.50±1.96 ^*^
**Ch-DHA**	9.00±1.03
**HEK-CM**	10.66±0.76

**Table 6.** Cognitive performance of mice groups showing visits to novel object in the NORT.

Groups (n=6 / group)	Number of visits to novel object Mean ± SEM
**NAC**	9.16±0.74
**HIHEK-CM**	7.00±1.09
**EE**	13.33±0.95
**Ch-DHA**	14.50±1.60 ^#^
**HEK-CM**	16.66±0.84 ^@@@^

## Discussion

### Spatial learning and memory

The present study results reveal that normal aging in mice is associated with cognitive and memory impairments, particularly in spatial learning. Aged mice supplemented with Ch-DHA demonstrated improved spatial learning, reduced memory errors, and better performance in choosing baited arms during the radial arm maze task. HEK-CM treatment also yielded positive outcomes, surpassing Ch-DHA supplementation on the 4 ^th^ trial day. EE exposure demonstrated benefits, particularly on the 4 ^th^ trial day, outperforming NAC-treated mice. Overall, Ch-DHA, HEK-CM, and EE interventions exhibited potential in mitigating spatial memory deficits in aging mice. High consumption of Ch during the perinatal period showed neuroprotective effect in animal models including during age related neuronal dysfunction. Perinatal Ch supplemented rats showed improvement in spatial memory performance and showed less errors relative to the untreated group when subjected to a 12-arm radial maze task. ^31^^,^^32^ Rats which were supplemented with Ch as infants were able to retain a larger number of items in working memory during their adulthood. ^33^ Studies also report that, dietary DHA supplementation enhances spatial memory in DHA deficient rats. ^34^ Recent studies observed that, combined supplementation of both Ch and DHA was more efficacious in enhancing hippocampal neurodevelopment. Prenatal supplementation of Ch and DHA showed significant improvement in spatial learning and other cognitive functions during adolescence. ^35^^,^^36^ Supplementation of Ch and DHA reduced memory errors in obese rats when subjected to eight arm radial maze test. ^37^ The hippocampus plays a major role in memory function. Hippocampal damage disturbs the memory consolidation. ^38^ Cell therapies are beneficial in restoring spatial learning in animal models of hippocampal degeneration. ^39^^,^^40^ Conditioned media which are derived from cell cultures are known to have excellent sources of neurotrophic factors and cytokines. ^41^^,^^42^ Furthermore, HEK-CM contain a neurotropic factor erythropoietin and other cytokines. ^43^^,^^44^ In kainic acid induced hippocampal damaged mice, HEK-CM treatment showed significant increase in neuro-protection and improvement of hippocampal cognitive function. ^45^^,^^46^ Further, previous studies in aged animals also showed that EE prevents age-associated impairments in spatial learning and cognition. ^26^^,^^47^

### Cognition

In the present study, HEK-CM-treated aging mice showed increased preference for both familiar and novel objects, surpassing NAC and HIHEK-CM groups. Ch-DHA supplementation led to higher visits to novel objects, while EE-exposed mice exhibited a significant preference for familiar objects. These results highlight the varied effects of interventions on novel object recognition in aging mice.

Studies show that kainic acid (KA) lesioned mice that received HEK-CM showed a significantly greater number of visits towards the novel object as compared with KA alone treated mice. Normal mice that received HEK-CM also showed higher preference for novel object as compared with familiar objects. ^46^ Combined supplementation of nutrients like Ch and DHA after perinatal brain injury in mice showed a substantial preference for the novel object when subjected to NORT, indicating that recognition memory after brain injury was improved by the supplementation of DHA and Ch. ^48^ Aged mice, exposed to standard environment rather than EE, were able to discriminate between familiar and novel objects. Animals exposed to EE were able to distinguish stationary from displaced objects with different preferences independent of age. It was observed that young mice spent more time with displaced objects and aged mice with stationary objects. ^49^

Previous studies have detailed a few of the molecular mechanisms underlying the relationship between these strategies and their role in learning, memory, and cognition. Choline is known for its ability to enhance cholinergic function, while DHA, as an essential nutrient, facilitates neuronal development and processes such as neurogenesis, synaptogenesis, and synaptic plasticity in the hippocampus, ultimately enhancing cognitive capabilities. ^48^ Additionally, the HEK-CM is an excellent source of neurotrophic factors and contains known neuroprotectants such as erythropoietin and other cytokines that enhance cognition. ^44^^–^^46^ Exposure to EE has been found to stimulate neurogenesis, enhance the granule cell layer, and increase the number of dentate gyrus granule cells in the hippocampus. Moreover, EE exposure elevates the cAMP response element-binding protein (CREB) level in the hippocampus of aged animals. ^27^^,^^47^ Hence, it is evident that these strategies employ various underlying mechanisms to regulate both the structure and function of the hippocampus and related areas involved in cognitive processing. This variability could potentially account for the differences in behavioral outcomes observed in the present study in mice following exposure to these distinct age-associated cognitive-enhancing strategies. The current study might not capture the long-term effects of the interventions and sustained cognitive improvements. Although it assesses the comparative benefits of various interventions, further research is essential to unravel the molecular or cellular mechanisms, thereby enhancing the therapeutic approach.

## Conclusion

During normal aging in mice, supplementations of Ch-DHA and HEK-CM treatment strategies have a higher potential [~ 20—30%] for enhancing spatial learning, memory and cognition, whereas exposure to EE seems to enhance only short-term memory. Further studies need to be done in order to analyse the underlying mechanisms for the cognitive changes.

## Author contributions

Kiranmai S Rai and Anandh Dhanushkodi conceptualized and designed the methodology for this study. They have worked as project administrators and mentors/supervisors. The study was carried out in detail and investigated by Shreevatsa Bhat M. Data collection, curation and formal analysis of the data were carried out by Shreevatsa Bhat M. Resources were provided by Manipal Academy of Higher Education and partly by Anandh Dhanushkodi and Kiranmai S Rai. Shreevatsa Bhat M and Kiranmai S Rai have analysed and validated data. Shreevatsa Bhat M has written original draft. It has been critically revised and suggestions for editing were given by Kiranmai S Rai, Anandh Dhanushkodi and Ramesh Babu MG. Shreevatsa Bhat M, Ramesh Babu MG, Anandh Dhanushkodi and Kiranmai S Rai read the final version of article and have approved this version to be published.

## Data Availability

Dryad: Data for Radial arm maze tests and Novel object recognition test, https://doi.org/10.5061/dryad.xpnvx0kj3. ^50^ This project contains the following underlying data: File 1. Percentage_of_correct_choices-Learning_phase.csv (Percentage of correct choices made by mice during learning phase of eight arm radial maze test) File 2. Working_memory_error.csv (Number of working memory errors made by mice during trial phase of eight arm radial maze test) File 3. Reference_memory_error.csv (Number of reference memory errors made by mice during trial phase of eight arm radial maze test) File 4. Percentage_of_correct_choices-Retention_phase (Percentage of correct choices made by mice during retention phase of eight arm radial maze test) File 5. Visits_to_familiar_object.csv (Number of visits to familiar object made by mice during the test phase of novel object recognition test) File 6. Visits_to_novel_object.csv (Number of visits to novel object made by mice during the test phase of novel object recognition test) Dryad: ARRIVE checklist for ‘Exploring potential strategies to enhance memory and cognition in aging mice **’.** https://doi.org/10.5061/dryad.xpnvx0kj3. ^50^ Data are available under the terms of the Creative Commons Zero “No rights reserved” data waiver (CC0 1.0 Public domain dedication).
