# Supplementary material for: Exploring potential strategies to enhance memory and cognition in aging mice
Source: F1000Res. 2026 Jun 25;12:141. Originally published 2023 Feb 7. [Version 4] doi: 10.12688/f1000research.121922.4 (PMC13329362; doi:10.12688/f1000research.121922.4)
Supplement: Peer review history

# Reviewer report: Reviewer response for version 4

**DOI:** 10.5256/f1000research.204117.r497907

**Published:** 2026-07-02

**Authors:** David V C Brito

**Affiliations:** 1 Faculty of Medicine and Biomedical Sciences (FMCB), University of Algarve, Portugal, Portugal; 2 ABC Collaborative Laboratory: Integrated Ageing and Rejuvenation Solutions (ABC CoLAB – Ageing Better), Portugal, Portugal; 3 ABC-RI, Algarve Biomedical Center Research Institute, Faro, Portugal

Bhat et al. investigate the effects of three potential memory-enhancing interventions on behavioural measures of memory in middle-aged/aging male CF1 mice. Spatial learning and memory were assessed using the eight-arm radial maze, while recognition memory was assessed using the novel object recognition test. The authors report that Ch-DHA and HEK-CM improved performance in selected radial maze readouts and increased novel object visits in the NORT, whereas environmental enrichment showed more limited effects.

 The topic is relevant and the comparison of nutritional, cell-derived and environmental interventions in the same experimental framework has academic merit. The manuscript has improved compared with previous versions, particularly with the inclusion of additional methodological details, a clearer experimental scheme and a more explicit limitations section. The availability of the underlying behavioural data is also a strength.

 However, I still have several reservations regarding the strength of the conclusions. The main issues relate to the terminology and characterization of HEK-CM, the statistical analysis of repeated behavioural measures, the interpretation of the NORT data, the apparent mismatch in one of the figures, and the need to frame the findings more cautiously as exploratory.

**Major comments**

**1. **The manuscript repeatedly refers to “human embryonic kidney stem cell conditioned media”. However, the Methods section indicates that the conditioned medium was derived from cryopreserved 293T cells. HEK293T cells are an immortalized/transformed human embryonic kidney-derived cell line, not stem cells. Unless the authors can justify this terminology, I recommend replacing “human embryonic kidney stem cell conditioned media” with “HEK293T-conditioned medium” or “HEK cell-conditioned medium” throughout the whole manuscript.

 Moreover, the conditioned medium is insufficiently characterized in my opinion. For example, information on passage number, duration of conditioning, whether the medium was serum-containing at collection and whether protein concentration or any secreted factors were quantified are lacking. This might be important for the discussion of neurotrophic factors, erythropoietin, cytokines or other bioactive components are present.

**2. **The acquisition phase of the radial maze contains repeated measurements across days in the same animals. Therefore, analysing each day separately using one-way ANOVA does not fully capture the learning trajectory of each animal. A repeated-measures two-way ANOVA or mixed-effects model would be more appropriate for the acquisition data. This would allow the authors to determine whether the interventions truly altered learning over time rather than only producing group differences on individual days.

**3. **The current NORT analysis is based on the number of visits to the familiar and novel objects, analysed separately. This is a relatively indirect measure of recognition memory and may be influenced by general exploratory activity, locomotor behaviour, anxiety-like behaviour, or object-natural preference.

 Since the test was video monitored, I strongly recommend that the authors re-quantify the NORT data using exploration time. A more informative and standard readout would be the proportion of time spent exploring the novel object relative to total object exploration time, for example:

**Novel object preference = [time exploring novel object / (time exploring novel object + time exploring familiar object) ] x 100**

 Alternatively, the authors could calculate a discrimination index:

**Discrimination index = (novel object exploration time − familiar object exploration time) / total object exploration time**

 This would provide a more sensitive and interpretable measure of recognition memory. At minimum, the authors should report total object exploration time or total visits per group and discuss the limitations of using visit counts alone. This is particularly important because an increase in visits to the novel object may reflect increased overall exploration rather than improved recognition memory.

**4. **The manuscript states that HEK-CM and EE groups showed significantly greater visits to the familiar object. This does not straightforwardly indicate improved recognition memory. In standard NORT interpretation, preferential exploration of the novel object is usually taken as evidence of recognition of the familiar object. Increased familiar-object visits, especially in the absence of a discrimination index or total exploration analysis, may reflect altered exploration patterns rather than enhanced memory. The authors should revise this interpretation and avoid concluding that increased familiar object visits necessarily indicate improved cognition or short-term memory.

**5. **The figure labelled as showing visits to the familiar object in the NORT appears to display values and a y-axis corresponding to percentage of correct choices, rather than number of familiar-object visits. This does not match Table 5, where familiar-object visits are reported as approximately 5–12 visits. The authors should carefully check and correct Figure 6.

**6. **The data support cautious statements that Ch-DHA and HEK-CM were associated with improved performance in selected behavioural tasks. However, the current conclusion that these treatments have a higher potential for enhancing spatial learning and recognition memory by approximately 20–30% is too strong unless supported by direct statistical comparisons between intervention groups. The study does not establish therapeutic superiority or mechanism.

**Minor comments**

1. The environmental enrichment protocol should include additional details, including the number of animals per enriched cage, whether this differed from control housing, and whether object rotation followed a predefined schedule.
2. The authors should avoid mechanistic overstatements. References to BDNF, erythropoietin, neurogenesis, CREB, synaptic plasticity and anti-apoptotic pathways should be clearly presented as plausible mechanisms based on previous literature, not as mechanisms demonstrated in the present study.
3. The manuscript would benefit from further language editing. Some expressions remain imprecise for example: “lesser”, “spacial”, “mice species”, “normal aging mice”, and broad use of “cognition”.
4. The authors should consider adding individual data points to the graphs, especially given the small sample size.

Is the work clearly and accurately presented and does it cite the current literature?

Partly

If applicable, is the statistical analysis and its interpretation appropriate?

Partly

Are all the source data underlying the results available to ensure full reproducibility?

Yes

Is the study design appropriate and is the work technically sound?

Partly

Are the conclusions drawn adequately supported by the results?

Partly

Are sufficient details of methods and analysis provided to allow replication by others?

Partly

Reviewer Expertise:

aging, cognition, behavioral neuroscience, and preclinical mouse models.

I confirm that I have read this submission and believe that I have an appropriate level of expertise to confirm that it is of an acceptable scientific standard, however I have significant reservations, as outlined above.

# Reviewer report: Reviewer response for version 3

**DOI:** 10.5256/f1000research.159866.r293756

**Published:** 2024-07-05

**Authors:** Laibaik Park

**Affiliations:** 1 Weill Cornell Medical College, New York, NY, USA

Normal aging often leads to declines in various physiological functions, including cognitive abilities. Nutritional interventions have been reported to effectively prevent or delay age-related neuronal deficits, such as cognitive and behavioral declines. Nutrients like choline (Ch) and docosahexaenoic acid (DHA), along with therapies such as stem cell treatments and environmental enrichment (EE), have been shown to enhance brain function in humans and animals. However, there are no comparative studies on the neuroprotective effects of Ch, DHA, stem cells, and EE on age-related cognitive and memory declines. To address this gap, the authors treated male CF-1 mice (12-15 months old) with Ch-DHA, human embryonic kidney stem cell conditioned media (HEK-CM) and its control, and EE. Starting 30 days later, they conducted cognitive testing using an eight-arm radial arm maze and novel object recognition tests over 14 days. The authors report that Ch-DHA supplementation or HEK-CM treatment improved spatial learning, memory, and cognition, while EE was effective only in enhancing short-term memory. While the findings are intriguing, the manuscript is overly descriptive, unclear, and difficult to follow. Below are my suggestions to improve its quality.

 Majors,

1. The introduction clearly indicates that each of the treatments, such as Ch, DHA, HEK-CM, and EE, independently enhance brain function through unique mechanisms over varying treatment durations. For instance, maternal uptake of Ch during gestation increases nerve cell proliferation, DHA might improve cognitive function by increasing the level of synaptic proteins and membrane phospholipids in the hippocampal neurons, while cell and cell-derived therapies could enhance neural regeneration by chemical factors. EE might stimulate neuroplasticity through enriched environmental exposure. Based on these, it is uncertain whether it is feasible and informative to test and compare the effects of these treatments 30 days after their administration. The authors did not provide supporting data on each treatment. Authors should provide additional supporting data by conducting further experiments, such as Western blotting, immunohistochemistry, etc..
2. Although the authors claim that Ch-DHA supplementation or HEK-CM treatment improved spatial learning, memory, and cognition, with EE being effective only in enhancing short-term memory, the behavioral tests used (eight-arm radial arm maze and novel object recognition) are insufficient to support this claim. This is because the eight-arm radial arm maze is designed to assess working and reference memory, while the novel object recognition test evaluates recognition memory.
3. Only male mice were used in the study: why? Is it because the rearing environment affects radial-arm maze performance in male species, while its effect on females is inconsistent? Clear rationale and discussion are required.
4. Why was the treatment of Ch combined with DHA? Both treatments are known for their neuroprotective effects. Was it because pellet itself contained Ch content (1 mg/kg), as indicated on “Pellet feed …., Pune]” (page 4, lines 6-7)?  
5. In the animal section, the third line on page 10 indicates that 66 middle-aged (12 to 15-month-old) CF1 male mice were housed in the animal facility, but the first line of Experimental groups indicates that n=6/group was used: there is a discrepancy considering there were six treatment groups. In addition, was the power analysis performed to calculate the number of mice used for each group?
6. Were any proteins or neuroprotectants identified in the HEK-CM? Are there references supporting the experiments conducted with HEK-CM? If not, did the authors analyze the CM and assess its protein content?  
7. Including the experimental protocol as a figure could enhance the understanding of the study.
8. The first paragraph of “Cognition” section in Discussion on page 10 should be rewritten to be understandable on the basis of the rationale of the manuscript.
9. The second paragraph of the "Cognition" section in the Discussion on page 10 needs to be revised to better align with the findings presented in the manuscript.

Minors,

1. Page 3, line 23 (Cellular senescence … organism) seems non-sense.
2. On page 4, line 8, ad libitum and freely are redundant.
3. To remove olfactory cues, different ethanol concentrations of alcohol were used: 50% in eight-arm radial arm and 70% in novel object recognition. Is there any reason?
4. The y-axis of Figure 1 should be labeled with the full scale (e.g., 0-100) rather than a shortened range (e.g., 20-70), which exaggerates the treatment effects.
5. English proof-reading is recommended.

Is the work clearly and accurately presented and does it cite the current literature?

Yes

If applicable, is the statistical analysis and its interpretation appropriate?

Partly

Are all the source data underlying the results available to ensure full reproducibility?

Yes

Is the study design appropriate and is the work technically sound?

No

Are the conclusions drawn adequately supported by the results?

Partly

Are sufficient details of methods and analysis provided to allow replication by others?

No

Reviewer Expertise:

Alzheimer's disease, vascular dementia, blood flow regulation

I confirm that I have read this submission and believe that I have an appropriate level of expertise to state that I do not consider it to be of an acceptable scientific standard, for reasons outlined above.

# Response

**Published:** 2026-06-16

**Authors:** Prof Kiranmai S Rai

**Affiliations:** Manipal Academy of Higher Education, Manipal, India

**Response to Reviewer 3**

 We sincerely thank the reviewer for the careful review of our manuscript and for the constructive comments and suggestions. We have revised the manuscript accordingly, and our point-by-point responses are provided below.

**Major suggestions**

1. The introduction clearly indicates that each of the treatments, such as Ch, DHA, HEK-CM, and EE, independently enhance brain function through unique mechanisms over varying treatment durations. For instance, maternal uptake of Ch during gestation increases nerve cell proliferation, DHA might improve cognitive function by increasing the level of synaptic proteins and membrane phospholipids in the hippocampal neurons, while cell and cell-derived therapies could enhance neural regeneration by chemical factors. EE might stimulate neuroplasticity through enriched environmental exposure. Based on these, it is uncertain whether it is feasible and informative to test and compare the effects of these treatments 30 days after their administration. The authors did not provide supporting data on each treatment. Authors should provide additional supporting data by conducting further experiments, such as Western blotting, immunohistochemistry, etc..

**Response:** We thank the reviewer for this important observation. The purpose of the present study was to perform an initial behavioral comparison of different neuroprotective strategies in middle-aged mice under identical testing conditions. We agree that choline, DHA, HEK-conditioned media, and environmental enrichment likely act through distinct cellular pathways and time courses. The 30-day post-treatment assessment was intentionally selected to determine whether the benefits of these interventions persist beyond the immediate treatment period and to provide a common endpoint for comparison across all treatment groups. This approach allowed us to evaluate the durability of functional outcomes despite differences in the underlying mechanisms of action. Because this study was designed as a preliminary behavioral screening, molecular analyses such as Western blotting or immunohistochemistry were not included. We have now explicitly acknowledged this limitation in the revised discussion section and highlighted mechanistic studies as an important direction for future research.

  

1. Although the authors claim that Ch-DHA supplementation or HEK-CM treatment improved spatial learning, memory, and cognition, with EE being effective only in enhancing short-term memory, the behavioral tests used (eight-arm radial arm maze and novel object recognition) are insufficient to support this claim. This is because the eight-arm radial arm maze is designed to assess working and reference memory, while the novel object recognition test evaluates recognition memory.

**Response:** We agree with the reviewer that the behavioral tests used assess specific memory domains rather than global cognition. We have revised the manuscript to state that the interventions improved performance in tasks related to working, reference, and recognition memory, rather than making broad claims about overall cognition.

  

1. Only male mice were used in the study: why? Is it because the rearing environment affects radial-arm maze performance in male species, while its effect on females is inconsistent? Clear rationale and discussion are required.

**Response:** We thank the reviewer for this important comment. Male mice were selected for this study to minimize biological variability and to maximize the ability to detect the effects of choline-containing supplementation. Sex differences in choline metabolism have been reported, with females exhibiting enhanced endogenous choline synthesis due to estrogen-mediated activation of the phosphatidylethanolamine N-methyltransferase pathway. [DOI: 10.1096/fj.07-8227com] This mechanism can increase endogenous choline availability and may partially compensate for dietary choline requirements. In contrast, male mice rely more heavily on dietary sources of choline, making them generally more responsive to changes in choline intake. Therefore, the use of male mice provided a more sensitive experimental model for evaluating the effects of Ch-DHA supplementation on memory-related outcomes. We have added this rationale to the Methods section with supporting references and included the lack of female subjects as a limitation of the study in the revised Discussion section.

  

1. Why was the treatment of Ch combined with DHA? Both treatments are known for their neuroprotective effects. Was it because pellet itself contained Ch content (1 mg/kg), as indicated on “Pellet feed …., Pune]” (page 4, lines 6-7)?

**Response:** Choline and DHA were administered in combination based on prior evidence suggesting complementary and potentially synergistic roles in phosphatidylcholine synthesis, neuronal membrane formation, synaptic plasticity, and cognitive function. [DOI: 10.3390/nu11051125] We have clarified this rationale in the Introduction and added the relevant reference.

  

1. In the animal section, the third line on page 10 indicates that 66 middle-aged (12 to 15-month-old) CF1 male mice were housed in the animal facility, but the first line of Experimental groups indicates that n=6/group was used: there is a discrepancy considering there were six treatment groups. In addition, was the power analysis performed to calculate the number of mice used for each group?

**Response:** The number “66” reported in the Animals section was a typographical error. We apologise for the error. The correct total number of animals used in the study was 36, with n=6 per group across six experimental groups. This has now been corrected in the revised manuscript. Power analysis was not performed because this study was exploratory in nature. We have now acknowledged this as a limitation.

  

1. Were any proteins or neuroprotectants identified in the HEK-CM? Are there references supporting the experiments conducted with HEK-CM? If not, did the authors analyze the CM and assess its protein content?

**Response:** The conditioned media was used based on prior studies demonstrating neurotrophic effects. Previous studies have demonstrated neuroprotective potential of HEK-CM in animal models and in vitro neurodegenerative conditions attributing to endogenous upregulations of erythropoietin, BDNF and anti-apoptotic factors. [DOI: 10.1016/j.jcyt.2014.07.001 & DOI: 10.1155/2014/194967] We have added references supporting the biological relevance of HEK-conditioned media and clarified this in the discussion.

  

1. Including the experimental protocol as a figure could enhance the understanding of the study.

**Response:** We thank the reviewer for the suggestion. We have included a schematic diagram of the experimental protocol in the revised manuscript.

  

1. The first paragraph of “Cognition” section in Discussion on page 10 should be rewritten to be understandable on the basis of the rationale of the manuscript.
2. The second paragraph of the "Cognition" section in the Discussion on page 10 needs to be revised to better align with the findings presented in the manuscript.

**Response:** We thank the reviewer for this valuable suggestion. The first and second paragraphs of the Cognition section have been revised to improve clarity and better align the discussion with the study rationale and findings.

**Minor suggestions**

  

1. Page 3, line 23 (Cellular senescence … organism) seems non-sense.

**Response:** The sentence has been revised for clarity and scientific accuracy in the revised manuscript.

  

1. On page 4, line 8, ad libitum and freely are redundant.

**Response:** The sentence has been corrected in the revised manuscript.

  

1. To remove olfactory cues, different ethanol concentrations of alcohol were used: 50% in eight-arm radial arm and 70% in novel object recognition. Is there any reason?

**Response:** In both the eight-arm radial maze and novel object recognition test, 70% ethanol was used to remove olfactory cues between trials. The manuscript has been corrected accordingly.

  

1. The y-axis of Figure 1 should be labeled with the full scale (e.g., 0-100) rather than a shortened range (e.g., 20-70), which exaggerates the treatment effects.

**Response:** The y-axis of figure has been revised to display the full scale as suggested by the reviewer.

  

1. English proof-reading is recommended.

**Response: **The manuscript has been carefully proofread, and necessary grammatical, language, and typographical corrections have been made wherever applicable.

# Response

**Published:** 2026-06-16

**Authors:** Prof Kiranmai S Rai

**Affiliations:** Manipal Academy of Higher Education, Manipal, India

**Response to Reviewer 3**

 We sincerely thank the reviewer for the careful review of our manuscript and for the constructive comments and suggestions. We have revised the manuscript accordingly, and our point-by-point responses are provided below.

**Major suggestions**

 1. The introduction clearly indicates that each of the treatments, such as Ch, DHA, HEK-CM, and EE, independently enhance brain function through unique mechanisms over varying treatment durations. For instance, maternal uptake of Ch during gestation increases nerve cell proliferation, DHA might improve cognitive function by increasing the level of synaptic proteins and membrane phospholipids in the hippocampal neurons, while cell and cell-derived therapies could enhance neural regeneration by chemical factors. EE might stimulate neuroplasticity through enriched environmental exposure. Based on these, it is uncertain whether it is feasible and informative to test and compare the effects of these treatments 30 days after their administration. The authors did not provide supporting data on each treatment. Authors should provide additional supporting data by conducting further experiments, such as Western blotting, immunohistochemistry, etc..

**Response:** We thank the reviewer for this important observation. The purpose of the present study was to perform an initial behavioral comparison of different neuroprotective strategies in middle-aged mice under identical testing conditions. We agree that choline, DHA, HEK-conditioned media, and environmental enrichment likely act through distinct cellular pathways and time courses. The 30-day post-treatment assessment was intentionally selected to determine whether the benefits of these interventions persist beyond the immediate treatment period and to provide a common endpoint for comparison across all treatment groups. This approach allowed us to evaluate the durability of functional outcomes despite differences in the underlying mechanisms of action. Because this study was designed as a preliminary behavioral screening, molecular analyses such as Western blotting or immunohistochemistry were not included. We have now explicitly acknowledged this limitation in the revised discussion section and highlighted mechanistic studies as an important direction for future research.

 2. Although the authors claim that Ch-DHA supplementation or HEK-CM treatment improved spatial learning, memory, and cognition, with EE being effective only in enhancing short-term memory, the behavioral tests used (eight-arm radial arm maze and novel object recognition) are insufficient to support this claim. This is because the eight-arm radial arm maze is designed to assess working and reference memory, while the novel object recognition test evaluates recognition memory.

**Response:** We agree with the reviewer that the behavioral tests used assess specific memory domains rather than global cognition. We have revised the manuscript to state that the interventions improved performance in tasks related to working, reference, and recognition memory, rather than making broad claims about overall cognition.

 3. Only male mice were used in the study: why? Is it because the rearing environment affects radial-arm maze performance in male species, while its effect on females is inconsistent? Clear rationale and discussion are required.

**Response:** We thank the reviewer for this important comment. Male mice were selected for this study to minimize biological variability and to maximize the ability to detect the effects of choline-containing supplementation. Sex differences in choline metabolism have been reported, with females exhibiting enhanced endogenous choline synthesis due to estrogen-mediated activation of the phosphatidylethanolamine N-methyltransferase pathway. [DOI: 10.1096/fj.07-8227com] This mechanism can increase endogenous choline availability and may partially compensate for dietary choline requirements. In contrast, male mice rely more heavily on dietary sources of choline, making them generally more responsive to changes in choline intake. Therefore, the use of male mice provided a more sensitive experimental model for evaluating the effects of Ch-DHA supplementation on memory-related outcomes. We have added this rationale to the Methods section with supporting references and included the lack of female subjects as a limitation of the study in the revised Discussion section.

 4. Why was the treatment of Ch combined with DHA? Both treatments are known for their neuroprotective effects. Was it because pellet itself contained Ch content (1 mg/kg), as indicated on “Pellet feed …., Pune]” (page 4, lines 6-7)?

**Response:** Choline and DHA were administered in combination based on prior evidence suggesting complementary and potentially synergistic roles in phosphatidylcholine synthesis, neuronal membrane formation, synaptic plasticity, and cognitive function. [DOI: 10.3390/nu11051125] We have clarified this rationale in the Introduction and added the relevant reference.

 5. In the animal section, the third line on page 10 indicates that 66 middle-aged (12 to 15-month-old) CF1 male mice were housed in the animal facility, but the first line of Experimental groups indicates that n=6/group was used: there is a discrepancy considering there were six treatment groups. In addition, was the power analysis performed to calculate the number of mice used for each group?

**Response:** The number “66” reported in the Animals section was a typographical error. We apologise for the error. The correct total number of animals used in the study was 36, with n=6 per group across six experimental groups. This has now been corrected in the revised manuscript. Power analysis was not performed because this study was exploratory in nature. We have now acknowledged this as a limitation.

 6. Were any proteins or neuroprotectants identified in the HEK-CM? Are there references supporting the experiments conducted with HEK-CM? If not, did the authors analyze the CM and assess its protein content?

**Response:** The conditioned media was used based on prior studies demonstrating neurotrophic effects. Previous studies have demonstrated neuroprotective potential of HEK-CM in animal models and in vitro neurodegenerative conditions attributing to endogenous upregulations of erythropoietin, BDNF and anti-apoptotic factors. [DOI: 10.1016/j.jcyt.2014.07.001 & DOI: 10.1155/2014/194967] We have added references supporting the biological relevance of HEK-conditioned media and clarified this in the discussion.

 7. Including the experimental protocol as a figure could enhance the understanding of the study.

**Response:** We thank the reviewer for the suggestion. We have included a schematic diagram of the experimental protocol in the revised manuscript.

 8. The first paragraph of “Cognition” section in Discussion on page 10 should be rewritten to be understandable on the basis of the rationale of the manuscript.

 9. The second paragraph of the "Cognition" section in the Discussion on page 10 needs to be revised to better align with the findings presented in the manuscript.

**Response:** We thank the reviewer for this valuable suggestion. The first and second paragraphs of the Cognition section have been revised to improve clarity and better align the discussion with the study rationale and findings.

**Minor suggestions**

 1. Page 3, line 23 (Cellular senescence … organism) seems non-sense.

**Response:** The sentence has been revised for clarity and scientific accuracy in the revised manuscript.

 2. On page 4, line 8, ad libitum and freely are redundant.

**Response:** The sentence has been corrected in the revised manuscript.

 3. To remove olfactory cues, different ethanol concentrations of alcohol were used: 50% in eight-arm radial arm and 70% in novel object recognition. Is there any reason?

**Response:** In both the eight-arm radial maze and novel object recognition test, 70% ethanol was used to remove olfactory cues between trials. The manuscript has been corrected accordingly.

 4. The y-axis of Figure 1 should be labeled with the full scale (e.g., 0-100) rather than a shortened range (e.g., 20-70), which exaggerates the treatment effects.

**Response:** The y-axis of figure has been revised to display the full scale as suggested by the reviewer.

 5. English proof-reading is recommended.

**Response: **The manuscript has been carefully proofread, and necessary grammatical, language, and typographical corrections have been made wherever applicable.

# Reviewer report: Reviewer response for version 3

**DOI:** 10.5256/f1000research.159866.r241943

**Published:** 2024-03-21

**Authors:** Sareesh Narayanan Naduvil

**Affiliations:** 1 Department of Physiology, School of Medicine and Dentistry, AUC-UK Track, University of Central Lancashire, Preston, England, UK

Thanks for carefully addressing most of my comments and revising the paper accordingly. The paper is improved. I have no further comments to make.

Is the work clearly and accurately presented and does it cite the current literature?

Yes

If applicable, is the statistical analysis and its interpretation appropriate?

Yes

Are all the source data underlying the results available to ensure full reproducibility?

Yes

Is the study design appropriate and is the work technically sound?

Yes

Are the conclusions drawn adequately supported by the results?

Yes

Are sufficient details of methods and analysis provided to allow replication by others?

Yes

Reviewer Expertise:

Physiology, Neuroscience, Electromagnetic Biology, Learning and Memory, and Medical Education

I confirm that I have read this submission and believe that I have an appropriate level of expertise to confirm that it is of an acceptable scientific standard.

# Reviewer report: Reviewer response for version 2

**DOI:** 10.5256/f1000research.150959.r173936

**Published:** 2023-06-12

**Authors:** Sareesh Narayanan Naduvil

**Affiliations:** 1 Department of Physiology, School of Medicine and Dentistry, AUC-UK Track, University of Central Lancashire, Preston, England, UK

Title: Exploring potential strategies to enhance memory and cognition in aging mice

 In this paper, Shreevatsa et al., investigated the beneficial effects of potential memory-enhancing strategies like supplementation of choline (Ch) and docosahexaenoic acid (DHA) or administration of human embryonic kidney stem cell conditioned media (HEK-CM) or exposure to environmental enrichment (EE), in attenuating the cognitive impairments in aging mice. They demonstrate that all of these strategies have beneficial effects against normal aging-related cognitive impairments in mice with the highest potential being the supplementation of Ch-DHA and HEK-CM treatment compared to EE.

 General comments: This is an interesting research work. It is very evident that the authors have meticulously planned and executed this research work. The paper is well-written. However, I suggest the authors to consider looking into the following minor aspects to further improve the paper.

 Specific comments:

 Abstract: 

 This section is well written and all necessary information is included. However, the following minor editing’s can be considered.

- Please write 12 as ‘Twelve’ as the authors are starting that sentence with the number 12.
- Consider ending the same sentence as HEK-CM groups, instead of HEK-CM mice.

Introduction: 

 This section is well-written with the citation of relevant pieces of literature. The authors have stated the novelty of the work along with mentioning the rationale for choosing different potential memory-enhancing strategies. 

 Materials and methods:

 Animals:

- Consider writing… A total of 66 middle-aged…
- Statements pertaining to the CF1 mouse need citation. The rationale for selecting CF1 mouse can be included briefly in the introduction with relevant citations. Please consider rephrasing the sentence describing the ‘benefits of using this animal model’. 
- The experimental period as per this paragraph is 44 days, but the subsequent sections of the paper mention this as 30 days. Please correct this discrepancy. Otherwise, please explain what the authors meant by mentioning it as 44 days in this paragraph itself. 

Experimental groups:

- Please refer to my above comment on the experimental period. 
- Consider writing the last sentence… HEK-CM through intravenous (tail vein) injections…
- It would be nice to mention the exact days on which these injections were given to the mice. Something like; the 1-5th day or 10-15th day of the experimental period. This will be helpful while replicating this methodology by other researchers. 

HEK-CM derivation:

- I suggest the authors move the below sentence about HIHEK-CM from the discussion section to this section. 
- “HIHEK-CM used as vehicle control for HEK-CM, has all the protein constituents of HEK-CM that are inactivated by heat and hence has no effect on cognition”.
- If possible, authors can also describe how they have achieved this briefly as a part of the methodology.
- Environmental enrichment: I would suggest the authors to add a brief account of how an enriched environment was provided to the animals in this study. Otherwise, please provide a citation if the authors have used a published method without any modifications. 

Novel object recognition test:

 Please mention the location (object position) of the placement of novel and familiar objects. I mean, whether they were placed on the corners or more centrally in the open field? 

 Statistical analysis:

 The statistical tests used were adequate and appropriate for comparing the data across groups. 

 Results:

- Please consider editing the first sentence as; Aging mice supplemented ‘with’ Ch-DHA… 
- Please read the manuscript carefully and revise this accordingly in various sections.
- Usually, the discrimination index is reported while reporting the NORT data. If possible, it would be nice to look into this aspect too as the authors have got some interesting results in HEK-CM and Ch-DHA groups in novel object exploration. 
  The following article describes a method to calculate this manually; 
  Denninger JK, Smith BM, Kirby ED. Novel Object Recognition and Object Location Behavioral Testing in Mice on a Budget. J Vis Exp. 2018 Nov 20;(141):10.3791/58593. doi: 10.3791/58593. PMID: 30531711; PMCID: PMC6800058.

Discussion

- Please consider avoiding repeating results in this section.
- In view of published literature and the author’s current data, a plausible mechanism for a positive change in learning and memory in experimental groups can be proposed. This will significantly augment the discussion section. 
- Cognition: The first paragraph is a repetition of the results and can be modified accordingly. The third paragraph has a mention of a previous study but there is no citation provided. Please provide a reference. 
- As mentioned above, please do consider moving the following sentence to the methodology section. “HIHEK-CM used as vehicle control for HEK-CM, has all the protein constituents of HEK-CM that are inactivated by heat and hence has no effect on cognition”.
- In view of published literature and the author’s current data, a plausible neural mechanism for how the strategies used in this study helped in preserving or improving learning & memory/cognition in aging animals can be briefly discussed without much speculation. This will be helpful in evoking further research in this area. A description of a few limitations of the current study would also enrich the discussion section. 

Conclusion:

 The conclusion is concise and appropriately written.

Is the work clearly and accurately presented and does it cite the current literature?

Yes

If applicable, is the statistical analysis and its interpretation appropriate?

Yes

Are all the source data underlying the results available to ensure full reproducibility?

Yes

Is the study design appropriate and is the work technically sound?

Yes

Are the conclusions drawn adequately supported by the results?

Yes

Are sufficient details of methods and analysis provided to allow replication by others?

Yes

Reviewer Expertise:

Physiology, Neuroscience, Electromagnetic Biology, Learning and Memory, and Medical Education

I confirm that I have read this submission and believe that I have an appropriate level of expertise to confirm that it is of an acceptable scientific standard, however I have significant reservations, as outlined above.

## References

[1] : Novel Object Recognition and Object Location Behavioral Testing in Mice on a Budget. *J Vis Exp* .2018; 10.3791/58593 10.3791/58593 30531711 PMC6800058

# Response

**Published:** 2023-12-07

**Authors:** Prof Kiranmai S Rai

**Affiliations:** Manipal Academy of Higher Education, Manipal, India

The following (written in BOLD) provides brief details about the corrections and modifications made in the revised manuscript under different sections, in accordance with the suggestions from the reviewer.

 Materials and Methods:

**In the revised manuscript, the experimental and treatment periods have been clearly defined. The rationale for selecting mice has been moved to the introduction section from this part and cited appropriately, as suggested. Additionally, details about the vehicle control and HIHEK-CM have been modified based on suggestions and have been relocated from the discussion section to this part. The revised manuscript also provides specific information on how the environmental enrichment was administered. In response to the reviewer's query regarding object positioning during the novel object recognition test (NORT), the objects were centrally placed within the open field, ensuring an equal distance between each object and the sides of the field. This clarification has been incorporated into the revised manuscript.**

 Results:

**Minor corrections and modifications suggested have been incorporated in the result section. Regarding the analysis of the discrimination index in the NORT, we clarify that only the number of visits to the objects was counted during the test session, and due to the inability to record object exploration time, the discrimination index could not be reported. We are open and appreciative of these suggestions, and we will duly consider them in our ongoing and future research endeavours.**

 Discussion:

**The discussion section has undergone comprehensive modification and revision based on the provided suggestions. The limitations of the current study are also outlined in the end of the discussion section.**

# Reviewer report: Reviewer response for version 1

**DOI:** 10.5256/f1000research.133839.r164703

**Published:** 2023-03-09

**Authors:** Kumar M.R. Bhat

**Affiliations:** 1 Department of Anatomy, Ras Al Khaimah College of Medical Sciences, RAK Medical and Health Sciences University, Ras Al Khaimah, United Arab Emirates

The work is very well conceptualized and objectives are designed.  All sections of the article are well written. Results are encouraging further research in this area of age and cognition. 

 Abstract - sufficient

 Introduction - covers all necessary information to understand the objectives and its importance

 Methodology - overall content are good enough. However, few details about need to use of CF1 mouse would be important in this study.

 Result - good descriptions

 Discussion - can be enriched if the explain about:

1. molecular mechanism of Ch and DHA in relation to cognition
2. microconstituents of HEK media and its effect on cognition
3. possible mechanism about how EE works on cognition

Was there any signs of immune reactions due to HEK media? What are the difference between HEK-CM AND HIHEK-CM in terms of constituents and its effect on immune system and cognition?

 The author needs to make an attempt to explain the possible reasons for differences in the results after exposure to different enhancing agents.

Is the work clearly and accurately presented and does it cite the current literature?

Yes

If applicable, is the statistical analysis and its interpretation appropriate?

Yes

Are all the source data underlying the results available to ensure full reproducibility?

Yes

Is the study design appropriate and is the work technically sound?

Yes

Are the conclusions drawn adequately supported by the results?

Yes

Are sufficient details of methods and analysis provided to allow replication by others?

Yes

Reviewer Expertise:

Human gross anatomy, Neuroanatomy, neurobehaviour, Natural medicine and their mechanism of actions in human diseases, cancer biology, molecular biology.

I confirm that I have read this submission and believe that I have an appropriate level of expertise to confirm that it is of an acceptable scientific standard, however I have significant reservations, as outlined above.

# Response

**Published:** 2023-05-06

**Authors:** Prof Kiranmai S Rai

**Affiliations:** Manipal Academy of Higher Education, Manipal, India

Methodology -  However, few details about need to use of CF1 mouse would be important in this study.

**Answer: CF1mice were used as they genetically resembles human gene and also since these mice are smaller in size the total cost for the experiments was lesser.**

 Discussion - can be enriched if the explain about:

1. molecular mechanism of Ch and DHA in relation to cognition: **Choline is known to  enhance cholinergic function and DHA is an essential nutrient that helps development of neurons / neurogenesis, synaptogenesis / synaptic plasticity in hippocampus which enhances cognitive abilities**
2. microconstituents of HEK media and its effect on cognition- **HEK conditioned media  are excellent sources of neurotrophic factors and cytokines. Furthermore, the conditioned medium contains known neuroprotectants such as erythropoietin (EPO) and other cytokines. Previous studies show that these neurotrophic factors enhance cognition.**
3. possible mechanism about how EE works on cognition: **Previous studies revealed that exposure to EE has stimulating effect on neurogenesis, enhances granule cell layer and increases number of dentate gyrus granule cells in the hippocampus. Further, long-term EE exposure prevents age-associated cognitive impairment by increasing cAMP response element-binding protein (CREB) level in the hippocampus which potentially contributes to improved cognitive abilities such as learning and memory in aged animals.[Ref. -in revised version]**

Was there any signs of immune reactions due to HEK media? - **No**

 What are the difference between HEK-CM AND HIHEK-CM in terms of constituents and its effect on immune system and cognition?

**HIHEK-CM- used as vehicle control for HEK CM, has all the protein constituents inactivated by heat, hence has no effect on immune system and cognition**
